# Mechanisms underlying linear ubiquitination and implications in tumorigenesis and drug discovery

**DOI:** 10.1186/s12964-023-01239-5

**Published:** 2023-11-28

**Authors:** Jack Li, Sijin Liu, Shitao Li

**Affiliations:** 1 Department of Biosciences, Rice University, Houston, TX 77005 USA; 2 Research Center for Eco-Environmental Sciences, Chinese Academy of Sciences, Beijing, 100085 People’s Republic of China; 3 Department of Microbiology and Immunology, Tulane University, New Orleans, LA 70112 USA

**Keywords:** Linear ubiquitination, LUBAC, Cancer, Inflammation, NF-κB

## Abstract

Linear ubiquitination is a distinct type of ubiquitination that involves attaching a head-to-tail polyubiquitin chain to a substrate protein. Early studies found that linear ubiquitin chains are essential for the TNFα- and IL-1-mediated NF-κB signaling pathways. However, recent studies have discovered at least sixteen linear ubiquitination substrates, which exhibit a broader activity than expected and mediate many other signaling pathways beyond NF-κB signaling. Dysregulation of linear ubiquitination in these pathways has been linked to many types of cancers, such as lymphoma, liver cancer, and breast cancer. Since the discovery of linear ubiquitin, extensive effort has been made to delineate the molecular mechanisms of how dysregulation of linear ubiquitination causes tumorigenesis and cancer development. In this review, we highlight newly discovered linear ubiquitination-mediated signaling pathways, recent advances in the role of linear ubiquitin in different types of cancers, and the development of linear ubiquitin inhibitors.

Video Abstract

**Supplementary Information:**

The online version contains supplementary material available at 10.1186/s12964-023-01239-5.

## Background

Ubiquitination is a reversible protein post-translational modification by covalently conjugating ubiquitin, a small 76-amino acid protein, to lysine residues in the target protein [1]. Like methylation and acetylation, ubiquitination is also coded to regulate cellular functions through the coordinated actions of enzymes, including “writers” for conjugating ubiquitin to the substrate, “readers” for ubiquitin recognition and function execution, and “erasers” for ubiquitin removal [2, 3]. The ubiquitin writer is a sequential three-step enzymatic reaction system that is mediated by ubiquitin-activating enzymes (E1), ubiquitin-conjugating enzymes (E2), and ubiquitin ligases (E3). There are 2 E1 enzymes, 30–50 E2 enzymes, and over 600 human E3 ligases in the human genome [4]. The diversity of substrate-binding E3 ligases codes various types of polyubiquitination featured with distinct polyubiquitin linkages. Each ubiquitin code, such as the unique polyubiquitin linkage, is recognized by a different reader, leading to a specific fate of ubiquitinated proteins and distinct functional consequences. For example, lysine 48 (K48)-linked polyubiquitin is recognized by 26S proteasome, resulting in protein degradation. Similarly, the conjugated polyubiquitin is removed by erasers, a group of enzymes known as deubiquitinases (DUBs), providing the recycling of ubiquitin into the cytosolic pool and a counterbalance to the ubiquitin-mediated signaling pathways.

Ubiquitination is critical for cell homeostasis and plays a vital role in many physiological processes, including protein degradation, signal transduction, DNA repair, cell proliferation, and immune response. Mounting evidence highlights the disruption of ubiquitin code in aberrant cell signaling and disease development and progression, including various types of cancers. For example, alterations in the activity of many E3 ligases and DUBs are significantly associated with the etiology of human malignancies [5]. There are many excellent reviews on the role of well-known ubiquitin codes, like K48- and K63-linked polyubiquitin, in cancers [5–7]. Recently, a new ubiquitin code, the N-terminally Methionine 1-linked linear polyubiquitin, has been found. Although linear ubiquitin is much less abundant than other types of ubiquitin chains, recent studies have found that they play pivotal roles in tumorigenesis and cancer pathogenesis. However, there is no comprehensive review of the emerging role of linear ubiquitination in cancer. In this review, we focus on the role of linear ubiquitin in cancers, delineate linear ubiquitin-mediated signaling pathways, and discuss current therapeutic approaches that target linear ubiquitination for cancer therapy.

### Linear ubiquitination process and regulation

**Fig. 1:**
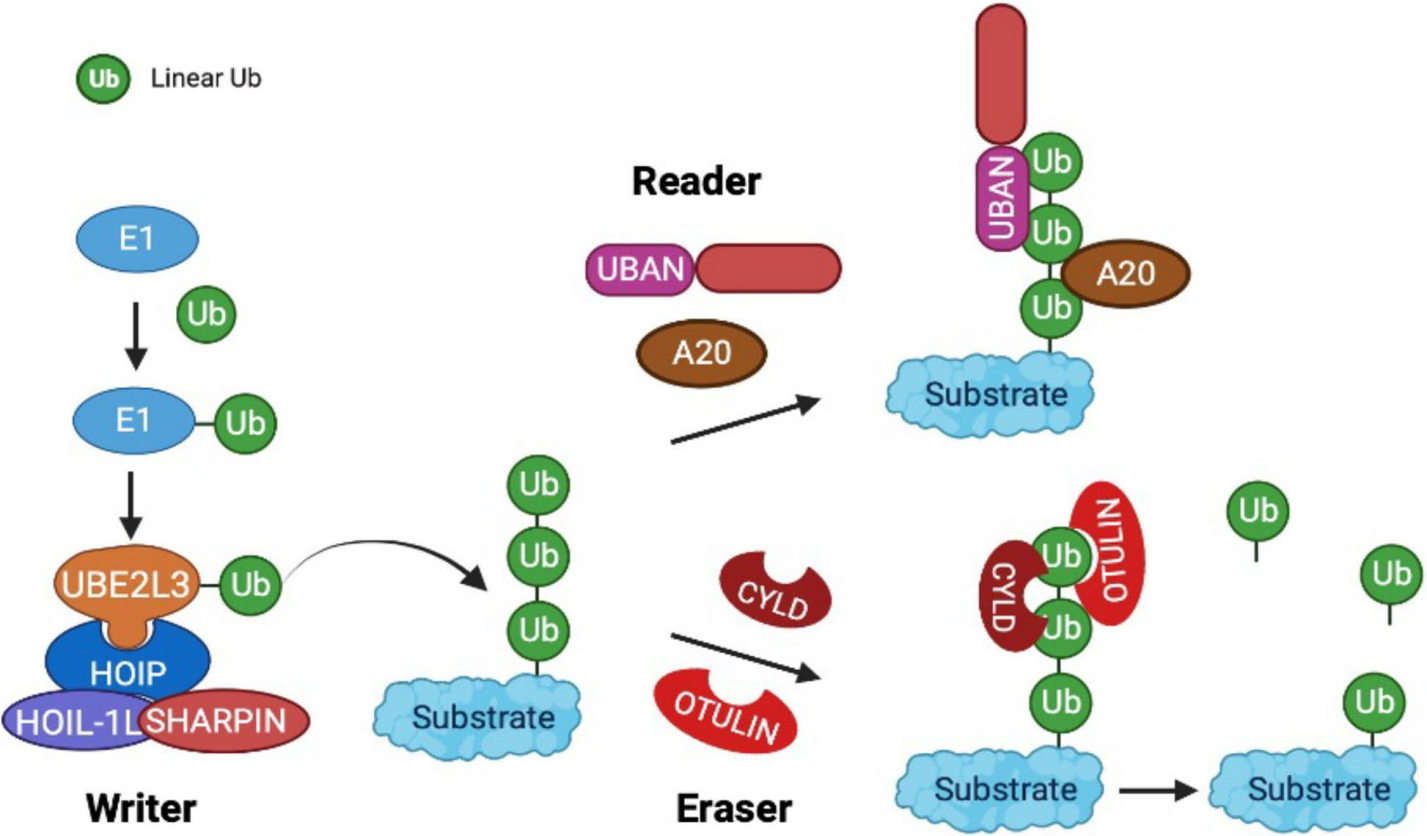
Schematics of the writer, reader, eraser, and regulator for linear ubiquitination. Linear ubiquitin chains are assembled by the E3 ligase LUBAC along with the E2 UBE2L3. LUBAC comprises HOIP, HOIL-1, and SHARPIN, which serves as the linear ubiquitin writer. Several proteins decode linear ubiquitin by specifically binding to translate into a cellular effect (readers). Two deubiquitinases disassemble linear ubiquitin chains as the erasers. A20, TNF-inducible protein A20; CYLD, cylindromatosis; HOIL-1, heme-oxidized IRP2 ubiquitin ligase 1L; HOIP, HOIL-1-interacting protein; OTULIN, OTU domain-containing deubiquitinase with linear linkage specificity; SHARPIN, SHANK-associated RH domain-interacting protein; UBAN, ubiquitin-binding domain in ABIN proteins and NEMO; UBE2L3, ubiquitin-conjugating enzyme E2 L3. The figure was created with BioRender.com

Most types of polyubiquitin linkage are determined by the covalent bond formed between the C-terminal glycine residue of one ubiquitin molecule and a lysine residue on the previous ubiquitin molecule, such as the K48- and K63-linked ubiquitination. However, linear ubiquitination is a type of non-canonical linkage characterized by the head-to-tail linkage of ubiquitin molecules via the C-terminal carboxyl group of the donor ubiquitin and the N-terminal methionine of the acceptor ubiquitin. This results in the formation of a peptide bond in contrast to isopeptide formation via the linkage to the epsilon amino group of a lysine residue. This section discusses the writer, readers, erasers, regulators, and substrates for linear ubiquitination (Fig. 1)*.*

### Writer: the LUBAC complex

**Fig. 2:**
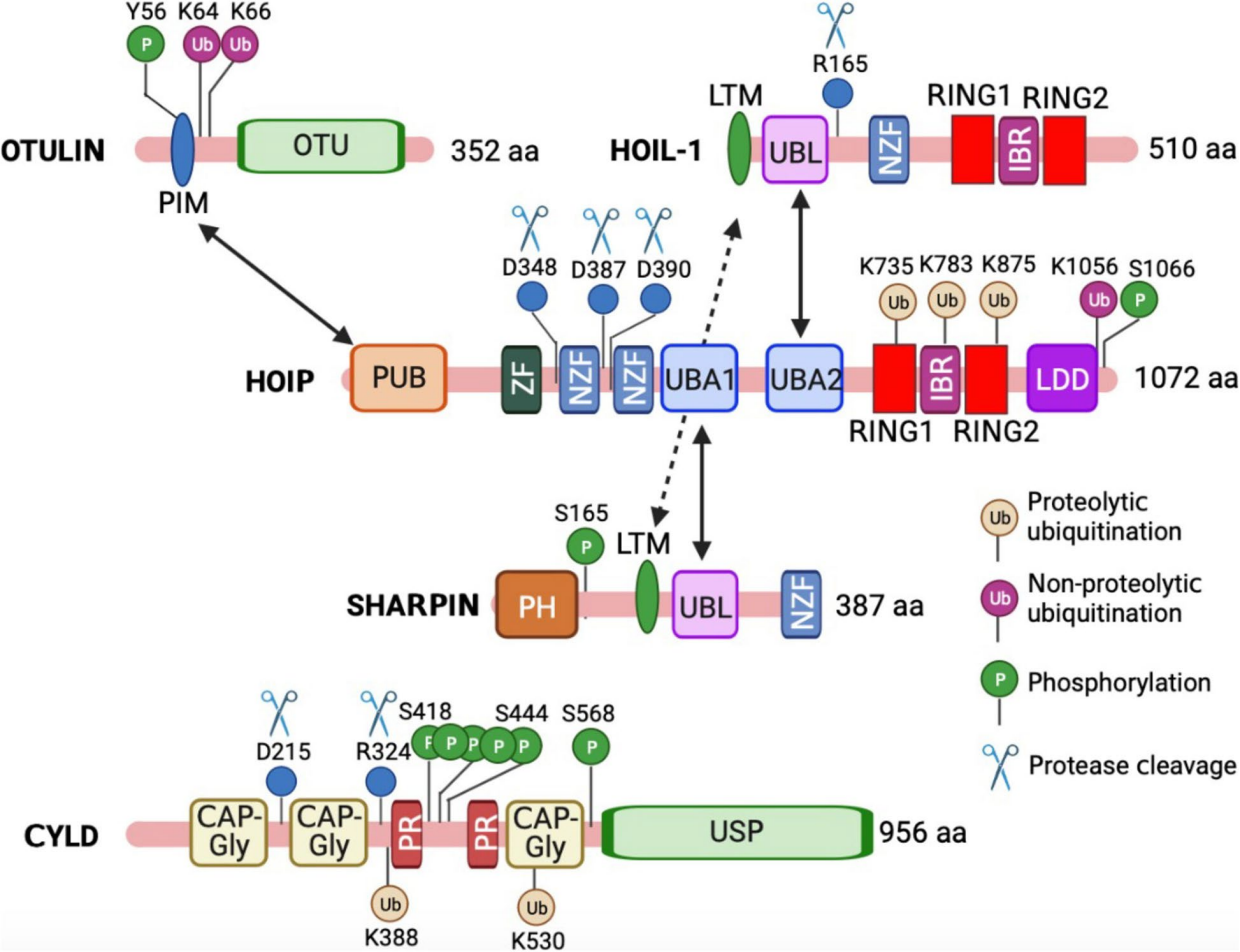
Domains and post-translational modification sites of the LUBAC subunits, OTULIN, and CYLD. CAP-Gly, cytoskeleton-associated protein glycine-rich; IBR, in-between RING; LDD, linear ubiquitin chain determining domain; LTM, LUBAC-tethering motif; OTU, ovarian tumor; PH, Pleckstrin-homology; PIM, PUB-interacting motif; PR, proline-rich region; PUB, PNGase/UBA or UBX; RING, really interesting new gene; UBL, ubiquitin-like; NZF, Npl4-type zinc finger; UBA, ubiquitin-associated; USP, ubiquitin-specific protease; ZF, zinc finger. The figure was created with BioRender.com

The writer for linear ubiquitin chains is the E3 ligase complex, the linear ubiquitin chain assembly complex (LUBAC) [2], along with the ubiquitin-conjugating enzyme E2 L3 (UBE2L3) [8]. LUBAC is a ~ 600 kDa complex consisting of three subunits, including two RING-between-RING (RBR)-type ubiquitin ligases, heme-oxidized IRP2 ubiquitin ligase 1L (HOIL-1L, also known as RBCK1) [9] and HOIL-1-interacting protein (HOIP, also known as RNF31) [10], and one adaptor protein SHANK-associated RH domain-interacting protein (SHARPIN) [11–13] (Fig. 2). Although HOIP and HOIL-1 are both E3 ligases, HOIP is the catalytically active component of LUBAC and the only E3 ubiquitin ligase that can assemble linear ubiquitin. HOIP recognizes ubiquitin-bound E2 at its RING1 domain and transfers ubiquitin from E2 to the active Cys885 in the RING2 domain via a thioester-linkage. Then, the C-terminal linear ubiquitin chain determining domain (LDD) facilitates the transfer of ubiquitin to the acceptor ubiquitin to form a linear linkage [14–17]. Interestingly, the catalytic activity of HOIP is autoinhibited by its N-terminal domain. The binding of the ubiquitin-like (UBL) domain of HOIL-1L and SHARPIN to the ubiquitin-associated domain (UBA) of HOIP releases HOIP from autoinhibition [14–16, 18, 19], suggesting that the integrity of LUBAC is critical for its activity. Furthermore, the E3 activity of HOIL-1L also regulates LUBAC activity. HOIL-1L conjugates monoubiquitin onto all LUBAC subunits, followed by HOIP-mediated conjugation of linear chains onto mono-ubiquitin, which attenuates the functions of LUBAC [20]. Overall, LUBAC is the sole E3 ligase complex that facilitates linear ubiquitination, which depends on the integrity of its three essential components.

### Readers

Ubiquitin chains are recognized by readers through their ubiquitin-binding domains. These readers further decode each type of ubiquitin linkage to execute specific functions. The readers for linear ubiquitin are UBAN (UBD in ABIN proteins and NEMO) domain-containing proteins [21] and A20 (also known as TNFAIP3) [22, 23]. The UBAN domain binds linear polyubiquitin and is shared by several proteins involved in NF-κB signaling pathways, including NF-κB essential modulator (NEMO), the A20 binding and inhibitor of NF-κB 1 (ABIN-1), ABIN-2, ABIN-3, and Optineurin (OPTN) [21]. Among these readers, the mechanism of how NEMO decodes linear ubiquitin is extensively studied. The UBAN of NEMO has a high affinity with linear ubiquitin via a surface on the proximal ubiquitin moiety and the canonical Ile44 surface on the distal one [24, 25]. The linear ubiquitin chain binding of NEMO recruits the IκB kinase (IKK) complex to the activation signalosome platform and induces IKK oligomerization and subsequent NF-κB activation [26]. A20 has a dual function as both DUB and E3 ligase due to the N-terminal OTU domain and a C-terminal zinc finger domain [27]. However, the DUB activity of A20 is not required for the NF-κB suppression. Instead, the interaction with linear ubiquitin through the ZF7 domain of A20 is crucial for NF-κB suppression [22, 23]. Thus, A20 is classified as a linear ubiquitin reader but not an eraser. In addition, HOIL-1L, the OTU domain-containing deubiquitinase with linear linkage specificity (OTULIN, also known as FAM105B or Gumby), and cylindromatosis (CYLD) are linear ubiquitin-binding proteins; however, their binding contributes to their roles in linear ubiquitin chain generation and removal, as discussed elsewhere. Collectively, NEMO and A20 are two extensively researched linear ubiquitin readers. Further exploration of proteins that bind to linear ubiquitin will likely uncover additional readers.

### Erasers: OTULIN and CYLD

The deubiquitinases, OTULIN and CYLD, bind to linear ubiquitin chains through their catalytic domains and hydrolyze linear polyubiquitin [28–34] (Fig. 2). OTULIN exclusively disassembles linear ubiquitin chains [28–30] while CYLD hydrolyzes both K63-linked and linear ubiquitin chains [32–34]. Furthermore, OTULIN but not CYLD prevents LUBAC from auto-ubiquitination [28, 30, 34, 35]. In addition, OTULIN knockout induces a strong increase in the abundance of linear ubiquitin [29, 36], which is not observed in CYLD-deficient cells [37], suggesting that these two erasers have distinct functions in the removal and regulation of linear polyubiquitin. A recent genetic study found that OTULIN promotes rather than counteracts LUBAC activity by preventing its auto-ubiquitination with linear polyubiquitin [35]. Although OTULIN and CYLD both are linear ubiquitin erasers, they have distinct phenotypes in vivo. While CYLD knockout mice show no major defects and are born at expected ratios [38], OTULIN null mutant mice (known as Gumby mice) are embryonic lethal [29]. These differences likely stem from their unique noncatalytic functions and interacting partners, which warrants further investigation in the future.

### Regulators

Recent studies show that the linear ubiquitin writer and erasers are tightly regulated by proteases and post-translational modifications, adding complexity to the different layers of linear ubiquitination. First, the two components of LUBAC, HOIP, and HOIL-1L, are cleaved by proteases. HOIP is cleaved at aspartate 348 (D348), D387, and D390 by caspases 3 and 6 during apoptosis [39, 40] and caspase 8 in the TNF-related apoptosis-inducing ligand (TRAIL)-induced cell death [41]. HOIL-1L is cleaved by paracaspase, mucosa-associated lymphoid tissue lymphoma translocation gene 1 (MALT1) [42–44]. MALT1 cleaves HOIL-1L between arginine 165 (R165) and glycine 166 (G166), resulting in the removal of the HOIL-1L RBR domain [44]. The RBR domain of HOIL-1L augments LUBAC activity, which is associated with the pathogenesis of B-cell-like diffuse large B-cell lymphoma [44, 45].

In addition to protease cleavage, HOIP is regulated by ubiquitination and phosphorylation. HOIP is ubiquitinated of K1056 at the carboxyl terminus by unknown E3 ligase(s) [46]. This ubiquitination dynamically alters HOIP conformation, resulting in the suppression of its catalytic activity. HOIP is phosphorylated at S1066 by the mammalian ste20-like kinase 1 (MST1), which attenuates the E3 ligase activity of LUBAC [47]. Similarly, SHARPIN is also a phosphorylated protein. SHARPIN is constitutively phosphorylated on serine 165 (S165) by an unknown kinase in lymphoblastoid cells. Moreover, this phosphorylation can be further enhanced by ERK1/2 kinases upon T cell receptor (TCR) engagement [48]. The S165A mutation of SHARPIN impairs the linear ubiquitination of NEMO and hinders NF-κB activation, suggesting that SHARPIN controls optimal activation of NF-κB response to both TCR and TNFα stimulation.

Like the ubiquitin writer, CYLD and OTULIN are also regulated by post-translational modifications. OTULIN activity is regulated by two post-translational modifications, phosphorylation and ubiquitination. OTULIN is phosphorylated at tyrosine 56 (Y56) located in the PUB domain-interacting motif (PIM). The PIM is responsible for binding to the N-terminal PUB domain of HOIP; however, the Y56 phosphorylation abrogates OTULIN-HOIP interaction [49–51]. Interestingly, OTULIN is hyper-phosphorylated at Y56 during necroptosis and counteracted by the dual specificity protein phosphatase 14 (DUSP14) [52]. Recently, Wang et al. reported that the ABL1 tyrosine kinase phosphorylates OTULIN at Y56 and promotes genotoxic Wnt/β-catenin activation to enhance drug resistance in breast cancers [53]. In addition, our lab recently found that OTULIN is subject to non-proteolytic ubiquitination upon TNFα stimulation [54]. We found that the E3 ligase tripartite motif-containing protein 32 (TRIM32) interacts with the OTU domain of OTULIN and conjugates non-proteolytic K63-linked polyubiquitin at K64 and K66 in the vicinity of the PIM domain. The polyubiquitin disrupts HOIP-OTULIN interaction, thereby enhancing TNFα-induced NF-κB activation [54].

CYLD protein stability is regulated by two different modifications. First, CYLD is subject to K48-linked ubiquitination and subsequent protein degradation. CYLD is ubiquitinated at K338 and K530 by the E3 ligase mind bomb homolog 2 (MIB2), which leads to CYLD proteasomal degradation and NF-κB activation [55]. CYLD is also targeted by another E3 ligase SCF^β−TRCP^ for degradation [56]. Second, CYLD is cleaved by proteases. It has been shown that caspase 8 (CASP8) cleaves CYLD at D215 in response to TNF and TLR stimulation [57, 58]. By contrast, upon TCR stimulation, the paracaspase MALT1 cleaves CYLD at arginine 324 (R324) [59]. In addition, CYLD is phosphorylated at the serine cluster between amino acids 418 and 444 by IKKα/β in response to TNF stimulation, which prevents CYLD from deubiquitinating the TNF receptor-associated factor 2 (TRAF2) and promotes TNF-induced gene expression [60]. Another IKK family member IKKɛ phosphorylates CYLD at S418, leading to decreased DUB activity [61]. By contrast, a recent study showed that IKK β phosphorylates CYLD at S569 to increase its DUB activity [62]. More importantly, S568 phosphorylation, in concert with S418 phosphorylation, switches CYLD’s DUB activity toward K63-linked polyubiquitin [63]. Furthermore, spermatogenesis-associated protein 2 (SPATA2) bridges CYLD to HOIP by binding to CYLD and HOIP via its PUB domain and PIM, respectively [64–67]. The SPATA2-mediated CYLD-HOIP interaction is essential for CYLD activity to linear ubiquitination and NF-κB activity [64–67].

Lastly, linear polyubiquitin itself is also regulated by post-translation modification. Wu et al. found that TNF induces the recruitment of LUBAC and the assembly of linear ubiquitin chains at mitochondria [68]. LUBAC stabilizes the PTEN-induced kinase 1 (PINK1) at the outer mitochondrial membrane by linear ubiquitination. PINK1 further phosphorylates linear ubiquitin at S65, which counteracts their OTULIN-mediated hydrolysis and augments NF-κB signaling [68].

Taken together, accumulating studies have shown that the writer and erasers of linear ubiquitin, as well as linear ubiquitin itself, is tightly regulated, which leads to distinct NF-κB signaling outcomes.

### Substrates

Many LUBAC substrates have been identified, including argonaute 2 (AGO2) [69], activin receptor-like kinase 1 (ALK1) [70], B cell CLL/lymphoma-10 (BCL10) [71], CASP8 [41], cellular FLICE-like inhibitory protein (cFLIP) [72], interleukin 1 receptor-associated kinase 1 (IRAK1) [73, 74], IRAK2 [73, 74], IRAK4 [73, 74], liver kinase B1 (LKB1) [75], myeloid differentiation primary response 88 (MyD88) [73, 74], NEMO [11, 76], PINK1 [68], phosphatase and tensin homolog (PTEN) [77], receptor-interacting protein kinase 1 (RIPK1) [11, 41], RIPK2 [30], signal transducer and activator of transcription 1 (STAT1) [78], STAT3 [79], and tumor necrosis factor receptor 1 (TNFR1) [30]. The linear ubiquitination sites and the roles of linearly ubiquitinated proteins are summarized in Table 1.

**Table 1:** Linearly ubiquitinated proteins and their functional roles

Gene	Ubiquitination site	Linear ubiquitination function	Ref
AGO2	K820	Interfering miRNA-targeted mRNA recruiting to AGO2	[69]
ALK1	K210, K229, K440, K491	Inhibiting ALK1 enzyme activity and Smad1/5 activation	[70]
BCL10	K17, K31, K63	Recruiting NEMO to activate IKK and NF-κB	[71]
CASP8	Not determined (n.d)	Probably required for TRAIL-induced NF-κB activation	[41]
cFLIP	K351, K353	Protecting cells from TNFα-induced apoptosis	[72]
IRAK1/2/4	n.d	Recruiting NEMO to activate IKK and NF-κB	[73, 74]
LKB1	n.d	Activating AMPK pathway to inhibit NLRP3 inflammasome response	[75]
MyD88	n.d	Recruiting NEMO to activate IKK and NF-κB	[73, 74]
NEMO	K285, K309	Required for TNFα- and IL1β-induced NF-κB activation	[11, 76]
PINK1	n.d	Stabilizing PINK1 protein and counteracting OTULIN activity	[68]
PTEN	K144, K197	Inhibiting PTEN phosphatase activity	[77]
RIPK1	n.d	Required for TNFα-induced NF-κB activation	[11, 41]
RIPK2	n.d	Augmenting NOD2-dependent proinflammatory signaling	[30]
STAT1	K511, K652	Inhibiting STAT1 binding to the type I IFN receptor IFNAR2	[78]
STAT3	K153, K161, K163, K199	Recruiting the phosphatase TC-PTP to STAT3 to reduce STAT3 activity	[79]
TNFR1	n.d	Probably required for NF-κB activation	[30]

### Linear ubiquitin signaling

**Fig. 3:**
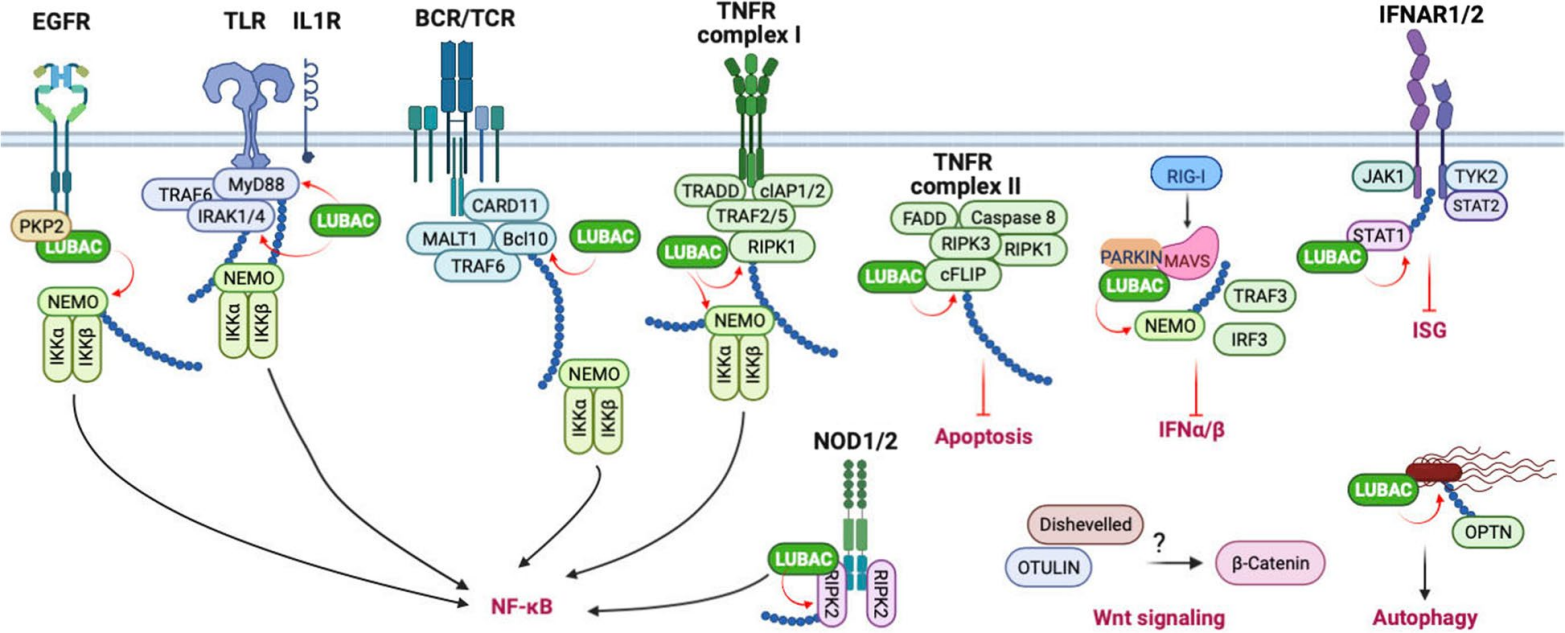
Linear ubiquitin-mediated signaling pathways. LUBAC catalyzes linear ubiquitin chains onto different substrates, and the linear ubiquitination leads to recruiting downstream signaling molecules, stabilizing target protein, and disrupting protein interaction. Each linear ubiquitin-mediated signaling pathway is discussed in detail in the main text. BCL10, the adaptor B cell CLL/lymphoma-10; BCR, B cell receptor; CARD11, scaffold caspase recruitment domain family member 11; cFLIP, cellular FLICE-like inhibitory protein; cIAP, cellular inhibitor of apoptosis; EGFR, epidermal growth factor receptor; FADD, FAS-associated death domain protein; IFN, interferon; IKK, IκB kinase; IL1R, interleukin 1 receptor type 1; IRAK, interleukin 1 receptor-associated kinase; IRF3, interferon regulatory factor 3; ISG, interferon-stimulated gene; JAK1, Janus kinase 1; MAVS, mitochondrial antiviral signaling protein; MyD88, myeloid differentiation primary response 88; NEMO, NF-κB essential modulator; NOD, nucleotide-binding oligomerization domain containing; OPTN, optineurin; PARKIN, Parkinson protein 2 E3 ubiquitin protein ligase; PKP2, plakophilin 2; RIG-I, retinoic acid-inducible gene I; RIPK, receptor-interacting protein kinase; STAT, signal transducer and activator of transcription; TCR, T cell receptor; TNFR, tumor necrosis factor receptor; TLR, Toll-like receptor; TRADD, TNFR1-associated death domain protein; TRAF, TNF receptor-associated factor; TYK2, tyrosine kinase 2. The figure was created with BioRender.com

Linear ubiquitin was first found to cue a signal in the TNFα- and IL1-instigated NF-κB pathways. Further studies have found that linear ubiquitin also plays a general role in many other NF-κB signaling pathways regulating innate and adaptive immunity, including IL-1, CD40, TRAIL, Toll-like receptors (TLRs), T and B cell receptors, EGF receptor (EGFR), nucleotide-binding oligomerization domain containing 1 (NOD1), and NOD2 receptors. However, recent studies suggest a diverse role of linear ubiquitin signaling beyond NF-κB signaling pathways. For example, linear ubiquitin suppresses the retinoic acid-inducible gene I (RIG-I)-mediated type I interferon (IFN) expression. Linear ubiquitin also participates in the Wnt signaling and autophagy process. All these exciting findings suggest that linear ubiquitin has more extensive roles than we thought before, which might attribute to its emerging role in cancer. These linear ubiquitin-mediated signaling pathways are discussed below (Fig. 3).

### Hybrid K63/Met1 polyubiquitin chain

In the MyD88 signaling pathways, including IL-1β and the TLR1/2 pathways, linear ubiquitin chains are usually covalently attached to K63-linked ubiquitin chains, which results in the formation of hybrid ubiquitin chains containing both types of linkage, known as K63/Met1-Ub hybrids [73]. The K63/Met1-Ub is known to conjugate to IRAK1 and IRAK4 in the MyD88 pathways [73]. Furthermore, K63/Met1-Ub is also found in the TNF, NOD1/2, and TLR3 signaling pathways, suggesting a general role of K63/Met1-Ub hybrids [80]. OTULIN can specifically hydrolyze the K63/Met1-Ub hybrids to free small K63-Ub oligomers of different lengths. Similarly, hydrolyzing K63-Ub chains releases small Met1-Ub oligomers of various lengths, suggesting a complicated topology of the K63/Met1-Ub hybrids. Although the structure of K63/Met1-Ub hybrid is yet to be solved, this hybrid form could facilitate IKK activation. As the TAB2 and TAB3 components of TAK1 complexes interact specifically with K63-Ub but not linear ubiquitin [32], the formation of K63/Met1-Ub hybrids may permit the recruitment of TAK1/TAB complex, thereby facilitating the TAK1-dependent activation of the IKK complex.

### NF-κB signaling pathways

Linear ubiquitination has been most extensively studied in the context of TNFα signaling. Upon TNFα engagement, TNFR1 trimerizes and forms a cytosolic signaling complex known as complex I through its cytosolic domain. The TNFR1 complex comprises E3 ligases, adaptors, and scaffold proteins, including RIPK1, cIAP1/2, and TRAF2/5. The K63-linked polyubiquitin in the TNFR1 complex recruits and activates the LUBAC. Then, the LUBAC complex conjugates linear ubiquitin chains onto NEMO and RIPK1 [11, 76, 81], which facilitates activation of TNFR1 signalosome. In addition, as discussed above, NEMO also binds to linear ubiquitin via its UBAN domain. Linear ubiquitination of NEMO promotes the oligomerization of the IKK complex and subsequent IKKα/β kinase activation. The activated IKK complex further phosphorylates the NF-κB inhibitor IκBα. Phosphorylation results in IκBα K48-linked ubiquitination and protein degradation, thereby releasing NF-κB heterodimers from their inhibitory binding and inducing the expression of NF-κB target genes.

Similarly, linear ubiquitin also regulates other NF-κB signaling pathways of innate immunity, including TLR (Toll-like receptor), interleukin-1 (IL-1), and TRAIL (TNF-related apoptosis-inducing ligand) pathways. Activation of IL-1R, TLR1/2, or TLR7/8 recruits the LUBAC complex to the myeloid differentiation primary response 88 (MyD88) activation complex. Then, LUBAC catalyzes the conjugation of linear chains to NEMO, IL-1 receptor-associated kinase 1 (IRAK1), IRAK2, IRAK4, and MyD88 [73, 74], thereby activating the IKK complex and downstream NF-κB signal cascades. Similarly, LUBAC also facilitates TRAIL-induced activation of NF-κB by promoting recruitment of the IKK complex [41].

In addition, TCR and B cell receptor (BCR) signaling also activate NF-κB via linear ubiquitination. TCR and BCR activation leads to the assembly of the CARD11 (scaffold caspase recruitment domain family member 11)-BCL10-MALT1 complex. LUBAC adds linear ubiquitin to BCL10 to promote NF-κB signaling [71]. However, genetic evidence found that deletion or reduction of HOIP abolishes NF-κB activation mediated by TCR and BCR, but LUBAC-mediated linear ubiquitination activity is dispensable [82–84]. Furthermore, the CD40- or the transmembrane activator and CAML interactor (TACI) -induced canonical NF-κB and ERK signaling pathways are impaired in B cells derived from HOIP knockout mice [82]. Whether and how linear ubiquitin is involved in these pathways needs further investigation.

### Inflammasome and NODosome

NOD-like receptors (NLRs) are a group of intracellular sensors that assemble into multi-protein complexes, such as the inflammasome and NODosome, upon activation. NLRPs represent the largest NLR subfamily and are characterized by the presence of an N-terminal Pyrin effector domain. During inflammasome activation, the activated NLRPs oligomerize and recruit adaptor proteins, including the apoptosis-associated speckled protein containing a CARD (ASC) and caspase 1, which leads to the cleavage of pro-IL-1 and pro-IL-18, and pyroptosis. A recent study found that HOIL-1L knockout mice have reduced IL-1β secretion in response to in vivo NLRP3 stimulation and survive the lethal challenge with LPS. A further mechanistic study showed that linear ubiquitination is required for NLRP3 inflammasome activation, suggesting a role of linear ubiquitin in inflammasome [85]. In contrast to inflammasome, NOD1/2 recruits RIPK2 to form a NODosome. LUBAC linearly ubiquitinates RIPK2 upon the ligand PGN stimulation, leading to activation of NF-κB and secretion of pro-inflammatory cytokines [86].

### Cell death

HOIP and HOIL-1 knockout mice are embryonic lethal due to massive cell death, suggesting that linear ubiquitination plays a critical role in the prevention of programmed cell death [11–13, 76, 87]. TNFα induces NF-κB activation through TNFR1 complex I as discussed above; however, it also triggers apoptosis and necroptosis through TNFR1 complex II consisting of RIPK1, RIPK3, FAS-associated death domain protein (FADD), caspase-8, and cFLIP. It has been speculated that linear ubiquitin chains play a role in stabilizing the TNFR1 complex I, leading to the inhibition of the formation of complex II and prevention of apoptosis [81]. However, a later study demonstrated that linear ubiquitination of RIPK1 is not essential for necrotic cell death [88]. Further study showed that LUBAC is required for TBK1 and IKKε recruitment to TNFR1 complex I in which TBK1/IKKε phosphorylates the kinase RIPK1 to prevent RIPK1-dependent cell death [89]. In addition, HOIP can directly interact with cFLIP and conjugate linear ubiquitin chains at K351 and K353 of cFLIP to stabilize cFLIP, thereby protecting cells from TNFα-induced apoptosis [72].

Linear ubiquitin also regulates TRAIL- and genotoxin-induced apoptosis. For example, LUBAC is recruited to TRAIL receptor-associated complex I and the cytoplasmic TRAIL-induced complex II. Following TRAIL stimulation, LUBAC linearly ubiquitinates RIPK1 and caspase 8 to prevent apoptosis and necroptosis [41]. In addition, LUBAC-mediated NEMO linear ubiquitination activates NF-κB and protects cells from genotoxin-induced apoptosis [90, 91].

### Type I IFN response

The attenuation of LUBAC activity in mice enhances type I IFN expression [35], suggesting an inhibitory role of linear ubiquitin in type I IFN production. Early studies showed that LUBAC inhibits the RIG-I-mediated IFN production signaling pathway by targeting TRIM25 for proteolytic ubiquitination and blocking TRIM25-RIG-I interaction [92]. It also has been shown that linearly ubiquitinated NEMO competes for TRAF3 binding with MAVS, a downstream signal molecule of RIG-I, thereby inhibiting type I IFN expression [93]. Hepatitis B virus induces PARKIN-dependent recruitment of LUBAC to the mitochondria and the unconjugated linear ubiquitin along with K63-linked polyubiquitin blocks MAVS-IRF3 interaction, thereby attenuating type I IFN expression [94]. However, other groups have shown that LUBAC activity is necessary for TLR3-mediated IFN response [95] and MDA5-mediated IFN expression induced by norovirus [96]. Thus, the role of linear ubiquitin in IFN induction is context-dependent, which warrants further investigation.

In addition, linear ubiquitin also regulates the type I IFN-elicited JAK-STAT signaling pathway. STAT1 is linearly ubiquitinated by LUBAC, and the conjugated linear ubiquitin inhibits the binding of STAT1 to the type I IFN receptor, IFNAR2, thereby blocking IFN-STAT1 signaling [78].

### EGFR signaling pathway

Epidermal growth factor receptor (EGFR) is a receptor tyrosine kinase that instigates several signaling cascades, including the NF-κB signaling pathway. Our recent study found that plakophilin 2 (PKP2) bridges LUBAC to the EGFR complex upon EGF stimulation [97]. The recruitment activated the LUBAC complex and the linear ubiquitination of NEMO, leading to IκB phosphorylation and subsequent NF-κB activation. Furthermore, EGF-induced linear ubiquitination was critical for tumor cell proliferation and tumor development [97].

### Wnt signaling pathway

By studying the Gumby mice, Rivkin et. al found that the OTULIN gene has missense mutations that are embryonic lethal due to vascular formation defects [29]. OTULIN interacts with Dishevelled, a key component of Wnt signal pathway to regulate β-catenin activity [29]. Furthermore, a recent study that the ABL1 tyrosine kinase phosphorylates OTULIN at Y56, leading to disruption of OTULIN-LUBAC interaction. This OTULIN phosphorylation promotes genotoxic Wnt/β-catenin activation and enhances drug resistance in breast cancers [53].

### Autophagy

Linear ubiquitin is involved in antibacterial autophagy (xenophagy) to restrict cytosol-invading bacteria, Salmonella Typhimurium. LUBAC is recruited to Salmonella by the ubiquitin coats on the bacteria [98–101]. LUBAC further generates linear ubiquitin chains, which are recognized by two linear ubiquitin readers, NEMO and OPTN [99]. OPTN is an autophagy adaptor that specifically interacts with linear ubiquitin chains through its UBAN motif. Thus, linear ubiquitin induces two signaling pathways, NEMO-mediated NF-κB activation and OPTN-induced xenophagy, thereby eliminating the bacteria [99].

### Protein quality control

p97/VCP is a triple A-type quality control ATPase that can extract ubiquitinated proteins from macromolecular complexes or lipid membranes. Like OTULIN, p97/VCP also has a PIM domain that is able to bind the PUB domain of HOIP [49–51]. It has been reported that HOIP is recruited to the aggregates derived from misfolded Huntingtin containing a pathogenic polyglutamine expansion (Htt-polyQ) through p97/VCP [102]. The recruitment of LUBAC results in the assembly of linear polyubiquitin on Htt-polyQ aggregates. Consequently, the interactive surface of misfolded Huntingtin species is shielded from unwanted interactions, such as the sequestration of low-complexity domain-containing transcription factors that cause transcriptional dysregulation in Huntington’s disease [102].

### Linear ubiquitin in cancer

Dysregulation of linear ubiquitination causes human diseases and pathologies. Early studies focus on human immunodeficiency and autoinflammation due to the role of linear ubiquitin in NF-κB signaling pathways. For example, mutations in HOIP and HOIL-1 cause human immunodeficiency and autoinflammation or polyglucosan storage myopathy and cardiomyopathy [103–106]. Genetic mutations in OTULIN cause the development of OTULIN-related autoinflammatory syndrome (ORAS), which is associated with recurrent fevers, autoantibodies, diarrhea, panniculitis, and arthritis [36, 107, 108]. Recently, linear ubiquitin-mediated NF-κB, cell death, and other signaling pathways have been found to play a crucial role in oncogenesis. In this section, we discuss the recent advances in the role of linear ubiquitination in different types of cancers (Table 2).

**Table 2:** Mechanisms underlying the role of linear ubiquitination in cancer

Cancer type	Gene	Mechanism	Ref
Lymphoma	HOIP	Q584H and Q662L mutations of HOIP increase LUBAC activity and NF-κB signaling	[45]
Lymphoma	CARD11	Oncogenic CARD11 variants facilitate BCL10 linear ubiquitination by co-recruitment of HOIP and BCL10	[71]
Myeloid leukemia	HOIP	Knockdown of HOIP increases apoptosis in myeloid leukemia cells	[109]
Breast cancer	Epsin1/2	Heightened epsin levels promote NEMO linear ubiquitination, resulting in sustained NF-κB signaling	[110]
Breast cancer	HOIP	HOIP facilitates p53 polyubiquitination and degradation by stabilizing MDM2	[111]
Breast cancer	SHARPIN	SHARPIN prolongs MDM2 protein stability to facilitate p53 degradation	[112]
Breast cancer	SHARPIN	SHARPIN mediates monoubiquitination of ERα, blocking ERα polyubiquitination and protein degradation	[113]
Breast cancer	HOIL-1	HOIL-1 promots the transcription of ERα and cyclin B1	[114]
Breast cancer	HOIL-1	HOIL-1 promotes YAP polyubiquitination and degradation	[115]
Prostate cancer	PTEN	R173H and R173C mutations of PETN enhance the linear ubiquitination of PTEN, inhibiting PTEN phosphatase activity	[77]
Ovarian cancer	HOIL-1	HOIL-1 promotes PTEN polyubiquitination and degradation	[116]
Renal cell carcinoma	HOIL-1	HOIL-1 facilitates p53 proteolytic ubiquitination	[117]
Renal cell carcinoma	SHARPIN	SHARPIN promotes the ubiquitination and proteasomal degradation of the tumor suppressor Von Hippel-Lindau	[118]
Skin cancer	SHARPIN	SHARPIN promotes Rap1 expression	[119]
Skin cancer	SHARPIN	SHARPIN enhances PRMT5 methyltransferase activity	[120]
Liver cancer	HOIL-1	HOIL-1 mediates the PPARγ/PGC1α complex ubiquitination and protein degradation	[121]
Liver cancer	OTULIN	OTULIN inhibits FADD and RIPK1 kinase-mediated hepatocyte apoptosis	[122]
Lung cancer	SHARPIN	SHARPIN interacts with PRMT5 to regulate histone H3R2 methylation-coupled transcriptional activation	[123]
Lung cancer	HOIL-1	HOIL-1 targets PKCζ for proteasomal degradation to promote tumor survival	[124]
Glioblastoma	OTULIN	OTULIN removes linear ubiquitin conjugated on STAT3, leading to persistent STAT3 signaling	[79]
Gastric cancer	SHARPIN	SHARPIN prevents β-catenin ubiquitination and proteasomal degradation	[125]

### Lymphoma

Diffuse large B-cell lymphoma (DLBCL) is the most frequent lymphoma subtype that is classified into two major categories, germinal center B-cell-like (GCB)-DLBCL and activated B-cell-like (ABC)-DLBCL. The ABC-DLBCL is characterized by constitutive NF-κB activation mediated by the B-cell receptor (BCR) and TLR signaling pathways. The BCR activation engages the CARD11-MALT1-BCL10 (CBM) adapter complex to activate the IKK complex and the canonical NF-κB pathway, which is the major pathogenic mechanism promoting malignant cell survival. Two germline missense single nucleotide polymorphisms (SNPs) in the HOIP gene are rare among healthy individuals (∼1%) but enriched in ABC DLBCL (7.8%) [45]. The two SNPs cause Q584H and Q662L mutations in the UBA domain of HOIP, resulting in the enhancement of HOIP and HOIL-1 interaction. Consequently, these mutations increase LUBAC activity and NF-κB signaling [45]. Furthermore, the mRNA expression of HOIP is elevated in human ABC-DLBCL [91], suggesting that there are multiple mechanisms promoting LUBAC activity in ABC-DLBCL.

In the mouse model, overexpression of HOIP increases LUBAC activity but fails to induce B-cell lymphoma. However, enforced HOIP expression facilitates the generation of B-cell lymphomas induced by oncogenic mutation of MyD88 [91]. In vitro analysis revealed that HOIP overexpression protected B cells from DNA damage-induced cell death through NF-κB activation [91].

Mechanistic studies further delineate the BCR-LUBAC signaling pathway. Upon BCR activation, the E3 ubiquitin ligases cIAP1/2 associated with the CBM complex assemble K63-linked polyubiquitin chains on themselves and on BCL10. These K63-linked ubiquitin chains recruit LUBAC and the IKK complex, which is essential for NF-κB activation [126]. In addition, oncogenic CARD11 variants associated with DLBCL, including C49Y, E93D, T112I, and G123D, spontaneously induce linear ubiquitination of BCL10 by co-recruitment of HOIP and BCL10 [71].

### Leukemia

Mouse knockout models suggest that LUBAC is required for fetal and adult hematopoiesis [109]. A recent mouse study found that HOIP is essential for myeloid leukemia propagation and maintenance [109]. The study utilized aggressive murine myeloid leukemia models driven by retroviral transduction of oncogenes: MLL-AF9 and NRAS G12V-driven acute myeloid leukemia (AML) and BCR-ABL and NUP98-HOXA9-driven blast crisis of chronic myelogenous leukemia (CML-BC) models. Deletion of HOIP impairs colony formation of leukemia cells in vitro, induces apoptosis in leukemia stem cells, and leads to significantly longer survival with reduced disease burden in the bone marrow and spleen. Furthermore, the knockdown of HOIP increases apoptosis in several myeloid leukemia cell lines and primary patient-derived AML cells. HOIP ligase activity and interaction with the other LUBAC subunits are essential for the growth of murine and human leukemia cells. These results indicate that HOIP is essential for the propagation and maintenance of murine and human myeloid leukemia [109].

### Breast cancer

A survey of LUBAC component expression found that the mRNA expression levels of all three subunits, HOIP, HOIL-1, and SHARPIN, are higher in breast cancer samples compared to adjacent non-tumor tissue [127]. The study also found that HOIP protein is significantly higher in estrogen receptor (ER)-negative tumors than in ER-positive tumors. ER-negative breast cancer exhibits elevated NF-κB activity and is more malignant and devastating than ER-positive breast cancer. A recent study found that two Epsin proteins, Epsin 1 and Epsin 2, interact with LUBAC and promote NEMO linear ubiquitination, resulting in the heightened IKK activation and sustained NF-κB signaling essential for the development of ER-negative breast cancer [110]. Elevated Epsin protein levels in ER-negative human breast cancer are also associated with poor relapse-free survival [110].

In addition, each component of LUBAC regulates breast cancer development through mechanisms independent of LUBAC activity. For example, HOIP associates with the p53/MDM2 complex and facilitates p53 polyubiquitination and degradation by stabilizing MDM2 in breast cancer cells [111]. Similarly, SHARPIN also binds to MDM2 and prolongs MDM2 protein stability to facilitate p53 degradation in breast cancer cells [112]. Whether HOIP and SHARPIN coordinate on MDM2 protein stability is an important question to be answered in the future. A recent study found that SHARPIN stabilizes ERα and promotes breast cancer cell proliferation. SHARPIN mediates monoubiquitination of ERα, which blocks ERα polyubiquitination and subsequent protein degradation [113].

HOIL-1 also regulates signaling pathways in breast cancer but in a more complicated way. It has been shown that HOIL-1 interacts with the HIF1α protein to indirectly inhibit its polyubiquitination and degradation, thereby promoting HIF1α-targeted gene expression as well as breast cancer progression [128]. HOIL-1 also drives breast cancer cell proliferation by promoting the transcription of ERα and cyclin B1 [114]; however, the underlying mechanism is not clear. By contrast, HOIL-1 mediates p65 ubiquitination and degradation, thus suppressing the NF-κB signaling pathway and tumorigenesis in breast cancer [129]. Furthermore, HOIL-1 associates with YAP protein and promotes YAP protein K48-linked polyubiquitination and degradation, thus inhibiting YAP-driven signaling and triple-negative breast cancer (TNBC) progression [115].

### Prostate cancer

Linear ubiquitin promotes prostate cancer (PCa) progression by enhancing AKT signaling in a PTEN-dependent manner [77]. PTEN is frequently mutated in human cancers, which leads to the excessive activation of PI3K/AKT signaling and thus promotes tumorigenesis and drug resistance. Specifically, LUBAC mediates linear ubiquitination of PTEN at two sites, K144 and K197, leading to inhibiting PTEN phosphatase activity and accelerating PCa progression. The study also found two high-frequency mutations of PTEN, R173H and R173C, in PCa patients who showed an enhanced linear ubiquitination of PTEN. Furthermore, HOIP is upregulated and positively correlated with AKT activation in PCa patient specimens, which may promote PCa progression and increase the risk of PCa biochemical relapse. In addition, high levels of SHARPIN are also found to associate with high malignancies and predicted shorter survivals of PCa patients [130].

### Ovarian cancer

Ovarian cancer (OC) is one of the most lethal gynecologic malignancies worldwide with a 5-year survival rate of less than 50%. An early study found that depletion of HOIP or expression of catalytically inactive HOIP sensitizes PEA1 ovarian cancer cells to genotoxin-induced apoptotic cell death [131]. By Oncomine™ analysis, they also revealed that HOIP and SHARPIN are overexpressed in serous ovarian carcinoma patient samples compared to normal tissue [131], suggesting a potential role of linear ubiquitin in OC. Most recently, it has been reported that PTEN is ubiquitinated in an OC cancer cell line SKOV-3 [116]. Unlike PTEN linear ubiquitination in prostate cancer discussed above, PTEN undergoes K48-linked ubiquitination and protein degradation, which is mediated by HOIL-1, in turn, inhibiting apoptosis and promoting cell proliferation [116].

### Renal cell carcinoma

Renal cell carcinoma (RCC) represents 2 to 3% of all cancers and is the tenth most common worldwide. Although the role of linear ubiquitination in RCC is not clear, recent studies showed that two LUBAC components, HOIL-1 and SHARPIN, are upregulated in human RCC samples and correlated with poor prognosis in RCC patients [117, 118]. However, HOIL-1 and SHARPIN regulate RCC through non-linear ubiquitination [117, 118]. For example, HOIL-1 interacts with p53 and facilitates p53 proteolytic ubiquitination, thereby promoting RCC cell proliferation [117]. Similarly, SHARPIN promotes the ubiquitination and proteasomal degradation of the tumor suppressor Von Hippel-Lindau protein (pVHL), resulting in the sustained activation of hypoxia-induced factor 2α (HIF-2α) [118]. Future work needs to elucidate how HOIL-1 and SHARPIN promote tumor suppressor degradation and whether linear ubiquitin also plays an important role in RCC.

### Skin cancer

Mice lacking SHARPIN develop chronic proliferative dermatitis, characterized by progressive epidermal hyperplasia, apoptosis of keratinocytes, and cutaneous inflammation [12, 132, 133], suggesting a critical role of SHARPIN in skin inflammation and keratinocyte cell death. Recent studies underpin the role of SHARPIN in skin cancer. There are three major types of skin cancers: basal cell carcinoma (BCC), squamous cell carcinoma (SCC), and melanoma. SHARPIN is downregulated or absent and highly mutated in cancer nests and precancerous BCC and SCC lesions compared with normal skin samples [134, 135]. Knockdown of SHARPIN in TE354.T cells, a human BCC cell line, enhanced tumor cell proliferation, possibly due to the increased phosphorylation of two transcriptional factors, c‑JUN and GLI family zinc finger 2 (GLI2) [134]. By contrast, higher expression of SHARPIN correlates with worse survival of melanoma [119]. SHARPIN promotes melanoma development via p38 and JNK/c-Jun pathways by upregulating Rap1 expression [119]. A recent study found a linear ubiquitin-independent role for SHARPIN in melanoma. SHARPIN interacts with the protein arginine methyltransferase 5 (PRMT5) to enhance PRMT5 methyltransferase activity, thereby promoting melanogenesis through the SKI/SOX10 regulatory axis [120]. Whether SHARPIN-mediated linear ubiquitination is involved in skin cancer needs further investigation.

### Liver cancer

Hepatocellular carcinoma (HCC) is a primary tumor of the liver and the fifth most common cause of cancer worldwide. A recent study reported that HOIP and HOIL-1 are highly expressed in HCC and the upregulation indicates poor clinical outcomes in patients with HCC, suggesting an oncogenic role of LUBAC in HCC [136]. Mechanistically, HOIL-1 interacted with HOIP and repressed its ubiquitination and proteasomal degradation, suggesting that an intact LUBAC complex and LUBAC-mediated linear ubiquitination play an important role in HCC. In addition, HOIL-1 also can promote invasion and metastasis of hepatocellular carcinoma by mediating the PPARγ/PGC1α complex ubiquitination and protein degradation [121].

Furthermore, two recent studies on OTULIN corroborate the role of linear ubiquitin in HCC. Liver-specific deletion of OTULIN in mice causes spontaneous steatohepatitis, fibrosis, and HCC, which is independent of TNFR1 signaling [137]. OTULIN also prevents liver inflammation and HCC by inhibiting FADD and RIPK1 kinase-mediated hepatocyte apoptosis [122].

### Lung cancer

Elevated SHARPIN expression has been found in human lung adenocarcinoma and squamous cell carcinoma compared with normal lung samples [123]. Similar to its role in melanoma, SHARPIN interacts with PRMT5 in lung cancer cells. The SHARPIN-PRMT5 complex plays biological roles in tumor progression and invasion via regulating a unique histone H3R2 methylation-coupled transcriptional activation in lung cancer cells [123]. In addition, HOIL-1 also plays a role in lung cancer by targeting PKCζ for proteasomal degradation to promote tumor survival [124].

### Other types of cancers

A recent study found that linear ubiquitination negatively regulates STAT3 activity through the recruitment of the phosphatase TC-PTP to STAT3. Preferential expression of OTULIN in glioblastoma stem-like cells (GSCs) removes linear ubiquitin conjugated on STAT3, leading to persistent STAT3 signaling, which maintains the stemness and self-renewal of GSCs [79]. Similarly, HOIL-1 contributes to chemoresistance and stemness in colorectal cancer [138]. In addition, SHARPIN is required for the invasiveness and malignant growth of gastric cancer cells in vitro and in vivo. Mechanistically, SHARPIN competes with the E3 ubiquitin ligase β-Trcp1 for β-catenin binding, thereby decreasing β-catenin ubiquitination levels to abolish its proteasomal degradation [125].

**Fig. 4:**
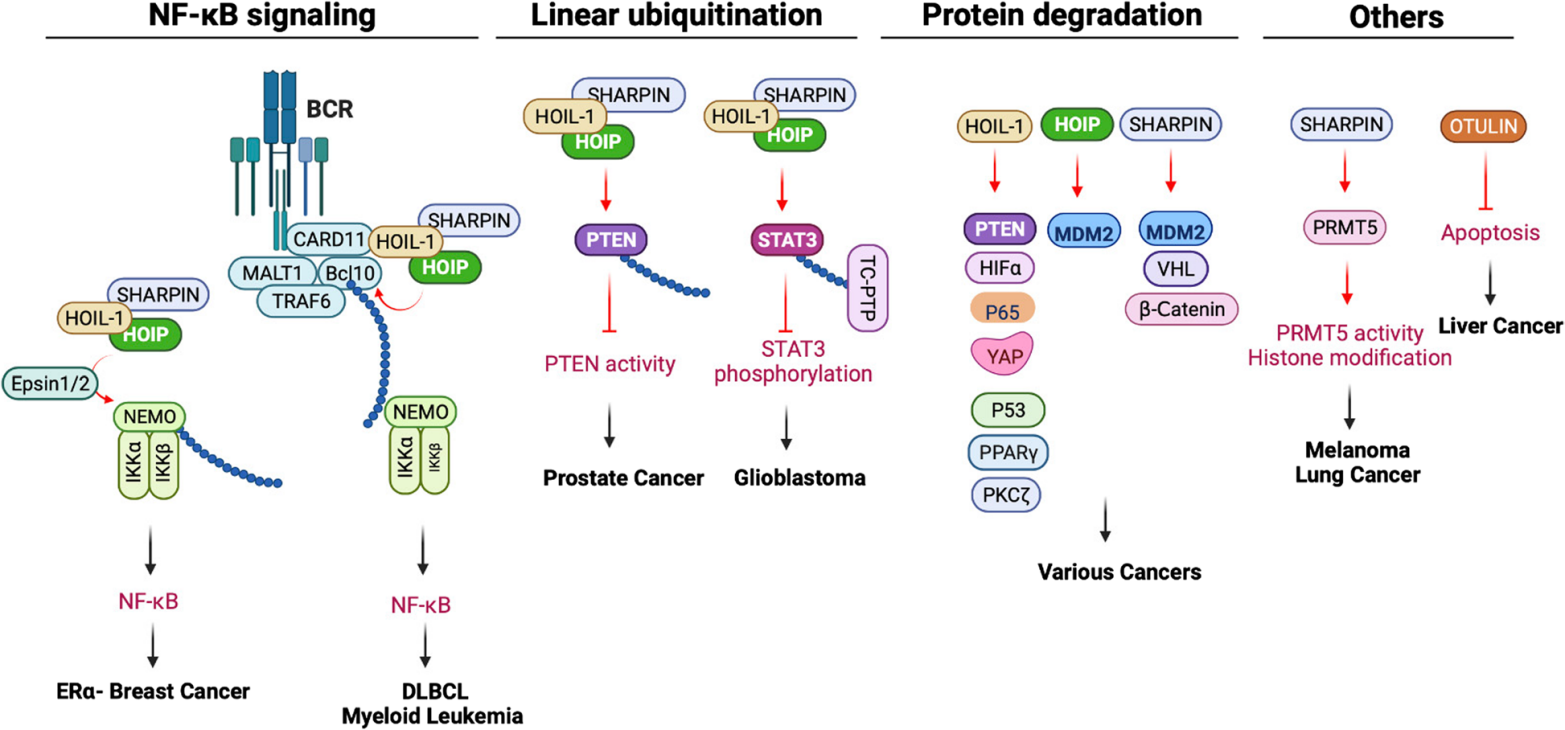
Mechanisms of linear ubiquitin and LUBAC in cancers. The mechanisms of LUBAC in cancers are roughly grouped into four types, as shown in the graph. ERα-, estrogen receptor-negative; DLBCL, diffuse large B-cell lymphoma; PTEN, phosphatase and tensin homolog; TC-PTP, T-cell protein tyrosine phosphatase; HIFα, hypoxia-induced factor α; YAP, Yes-associated protein; PPARγ, peroxisome proliferator-activated receptor γ; PKCζ, protein kinase C Zeta; MDM2, murine double minute 2; VHL, Von Hippel-Lindau protein; PRMT5, protein arginine methyltransferase 5. The figure was created with BioRender.com

Collectively, there are several common mechanisms for the role of LUBAC in cancers (Fig. 4). First, in ER-negative breast cancer and lymphoma, LUBAC-mediated NF-κB activation is the major driving factor. Second, LUBAC mediates the linear ubiquitination of proteins that do not participate in NF-κB signaling pathways, such as PTEN and STAT3. Linear ubiquitin regulates the enzymatic activity or activation of these proteins, ultimately promoting tumorigenesis. Third, each component of LUBAC can promote protein K48-linked ubiquitination and subsequent ubiquitination. Especially, HOIL-1 is also an RBR ubiquitin E3 ligase that plays a crucial role in various cancers by mediating the ubiquitination of a wide range of substrates. Last, LUBAC proteins play a regulatory role in the enzymatic activity of PRMT5 and histone methylation through interactions in melanoma and lung cancer, respectively. In addition, OTULIN prevents liver cancer by limiting apoptosis. The complex functions of LUBAC, which vary depending on the context, indicate that there might be intricate processes involved in various types of cancer. Therefore, further research is warranted for better understanding.

### Targeting LUBAC for cancer therapy

Linear ubiquitination attributes inflammation, acquired and innate immune responses, cell proliferation, chemoresistance, and metastasis in cancer [90, 131, 139]. LUBAC is the sole E3 that can generate a linear ubiquitin chain; thus, targeting LUBAC activity to reduce linear ubiquitination would be a promising therapeutic strategy for a broad spectrum of malignant tumors. Several natural and synthetic agents that inhibit LUBAC have been reported. Gliotoxin, the epipolythiodioxopiperazine metabolite from the marine fungus *Neosartorya pseufofischeri*, was the first small molecule shown to inhibit the linear ubiquitination activity of HOIP [140]. Gliotoxin inhibits LUBAC-mediated linear ubiquitination by binding to the catalytic center HOIP (aa. 699 − 1072). Gliotoxin inhibits TNFα-induced NF-κB activity [140] and sensitizes LSCC cells and mice to cisplatin [139]; however, gliotoxin also inhibits other proteins, such as NOTCH2 [141] and histone methyltransferase [142]. Thiolutin, a derivative of aureothricin, is also a natural product formed in submerged fermentation by *Streptomycetes*. Thiolutin inhibits LUBAC activity and suppresses the growth of B-cell lymphomas in a mouse transplantation model [91]. Like gliotoxin, thiolutin also has a broad-spectrum inhibition activity toward different substrates [143].

Besides natural products, synthetic small molecules have been tested for linear ubiquitination inhibition. For example, the IKK inhibitor BAY 11–7085 [144] has been found to inactivate LUBAC activity and prevent the formation of linear ubiquitin chains, which may contribute to its ability to induce B-cell lymphoma and leukemic T-cell death [145]. A high throughput screening based on E2 to E3 ubiquitin transfer activity identified bendamustine as a HOIP inhibitor. Bendamustine is an intravenously administered alkylating agent that perturbs DNA repair and cell cycle, resulting in cell death through apoptotic and nonapoptotic pathways [146]. Another high-throughput screening of a diverse library of electrophilic fragments identified two compounds, named (5) and (11a), as covalent binders of HOIP [147]. These compounds bind the RING2 domain of HOIP, which is not conserved in other RBR E3s. These sequence variations of RING2 might provide drug specificity to HOIP [147].

A recent study screened a small molecule library for LUBAC inhibitors and identified a thiol-reactive, α,β-unsaturated carbonyl-containing compound, sodium (E)-2-(3-(2-methoxyphenyl)-3-oxoprop-1-en-1-yl) benzoate, which is named HOIP inhibitor-1 (HOIPIN-1) [148]. The group further developed derivatives of HOIPIN-1, and found that sodium (E)-2-(3-(2,6-difluoro-4-(1H-pyrazol-4-yl)phenyl)-3-oxoprop-1-en-1-yl)-4-(1-methyl-1H-pyrazol-4-yl)benzoate, designated as HOIPIN-8, is the most potent LUBAC inhibitor [149]. HOIPINs are conjugated to the active site Cys885 in the RING2 domain of HOIP, thereby interrupting the RING-HECT-hybrid reaction within HOIP [150]. HOIPIN-8 effectively induces cell death in activated B cell-like diffuse large B cell lymphoma cells and alleviates imiquimod-induced psoriasis in model mice [150]. Furthermore, our lab showed that HOIPIN-8 inhibits EGFR-mediated NF-κB activation and cell proliferation of A431, MCF-7, and MDA-MB-231 cancer cells [97]. Most recently, Zhang et al. reported that HOIPIN-8 sensitizes colon carcinoma organoids to TNF and enhances bystander killing of MHC antigen-deficient tumor cells [151]. These studies suggest that HOIPIN-8 is a more specific LUBAC inhibitor and might be the most promising one for treating inflammatory diseases and cancer. Loss of LUBAC activity causes embryonic lethality in mice; thus, the in vivo toxicity of HOIPIN-8 needs to be investigated in the future.

In addition to the approach of drug screenings, rational drug design is also adopted. First, the LTM domain in HOIL-1L and SHARPIN associate with each other to form a globular domain, which is critical for the integrity and stability of LUBAC [18]. Thus, perturbing the LTM dimerization between HOIL-1 and SHARPIN would be a novel strategy to inhibit LUBAC. Indeed, an α-helical stapled peptide mimicking the LTM of SHARPIN blocks the HOIL-1L/SHARPIN interaction, destabilizes the pre-existing LUBAC complex, and inhibits the growth of ABC-DLBCL cells [18]. Similarly, HOIP-based stapled alpha-helical peptides were designed to inhibit LUBAC by disrupting the HOIL-1L-HOIP interaction [152]. These peptides impair cell viability and reduce NF-κB activity in cells. Secondly, a strategy using an antibody to block HOIP active site is also developed. By screening a large synthetic single-domain antibodies (single-dAbs) library against the RBR of HOIP, Tsai et al. successfully identified tight-binding dAbs that recognize the catalytic domain of HOIP and inhibit HOIP E3 ligase activity [153]. However, the effects of these dAbs on NF-κB activity and cancer cell proliferation have not been explored yet. Nonetheless, these innovative strategies provide new directions for LUBAC inhibitor development.

## Conclusions

This review discusses recent advances in linear ubiquitin research on cancer. The dysregulation of linear ubiquitination supports aberrant oncogenic signaling in a plethora of tumors. Although linear ubiquitination-mediated NF-κB activation plays a major role in lymphoma, diverse mechanisms are found in other cancers. For instance, hypoxia is the most prominent feature in solid tumors. A recent study found that hypoxia induces LUBAC interaction with AGO2 and subsequent AGO2 linear ubiquitination. The ubiquitinated AGO2 restrains miRNA-mediated gene silencing, thereby facilitating global mRNA accumulation in cancer cells [69]. Another study reported that LUBAC catalyzes linear ubiquitination of the kinetochore motor CENP-E. KNL1 acts as a receptor of linear ubiquitin chains to anchor the kinesin motor CENP-E at attached kinetochores in prometaphase and metaphase, thereby promoting accurate chromosome segregation [154]. This process is critical for cell fate because the missegregation of chromosomes can result in aneuploidy and cancer. Nonetheless, despite the increasing list of LUBAC substrates, we are still far away from understanding the role of linear ubiquitination in cancer development and progress.

Current studies mainly focus on the role of linear ubiquitination in inflammatory responses and cell death. Excitingly, the role of linear ubiquitination in adaptive immunity is now emerging, as acquired immune responses are crucial for the control of tumorigenesis and tumor development. The HOIP conditional knockout mice in B cells showed impaired B cell development and defective antibody responses to thymus-dependent and thymus-independent II antigens [82]. In addition, the ABIN1 protein has a UBAN domain that binds to linear ubiquitin chains with high affinity [155]. Loss of the ubiquitin-binding activity by D485N mutation of ABIN1 causes glomerulonephritis with a high titer of pathogenic autoantibodies in mice [156, 157]. Thus, a better understanding of the mechanisms underlying the deregulation of linear ubiquitination in innate and adaptive immunity is crucial for developing targeted therapies against cancer.

Developing chemotherapeutic agents against LUBAC is an attractive strategy that may inhibit multiple oncogenic pathways in tumors. In addition to the current strategy discussed above, the fast-growing protein-targeting chimeric molecules (PROTACs) technology could be a promising strategy for targeting LUBAC [158]. These PROTAC molecules are bifunctional and comprise an E3 ligase-recruiting moiety, a short linker in the middle, and a ligand that targets the substrate of interest, thereby placing target proteins in the proximity of E3 ligases for proteolytic ubiquitination and protein degradation [158]. Therefore, targeting the components of LUBAC for protein degradation by PROTAC may be a promising therapeutic intervention in various cancers herein described.

In conclusion, dysregulation of linear ubiquitination has been implicated in the development and progression of cancer. Understanding the role of linear ubiquitination in cancer will provide insights into potential therapeutic targets for treating the disease. Ongoing research in this area is essential for developing new cancer therapies.

## Data Availability

Not applicable.
